# α,β-Enone Borylation by Bis(Pinacolato)Diboron Catalyzed by Cu_3_(BTC)_2_ Using Cesium Carbonate as a Base

**DOI:** 10.3390/nano11061396

**Published:** 2021-05-25

**Authors:** Amarajothi Dhakshinamoorthy, Mercedes Alvaro, Abdullah M. Asiri, Hermenegildo Garcia

**Affiliations:** 1 School of Chemistry, Madurai Kamaraj University, Madurai 625 021, Tamil Nadu, India; 2 Departamento de Quimica, Universitat Politecnica de Valencia, Av. De los Naranjos s/n, 46022 Valencia, Spain; malvaro@qim.upv.es; 3 Center of Excellence in Advanced Materials Research, King Abdulaziz University, Jeddah 21589, Saudi Arabia; aasiri2@kau.edu.sa; 4 Instituto Universitario de Tecnologia Quimica (CSIC-UPV), Universitat Politecnica de Valencia, Av. De los Naranjos s/n, 46022 Valencia, Spain

**Keywords:** borylation, 2-cyclohexenone, heterogeneous catalysis, metal organic frameworks

## Abstract

Cu_3_(BTC)_2_ (BTC: 1,3,5-benzenetricarboxylate) as a heterogeneous catalyst in the presence of cesium carbonate as a base is reported for the borylation of α,β-conjugated enones by bis(pinacolato)diboron (B_2_pin_2_). According to the hot-filtration test, Cu_3_(BTC)_2_ is acting as a heterogeneous catalyst. Further, Cu_3_(BTC)_2_ exhibits a wide substrate scope and can be reused in consecutive runs, maintaining a crystal structure as evidenced by powder X-ray diffraction (XRD). A suitable mechanism is also proposed for this transformation using Cu_3_(BTC)_2_ as catalyst.

## 1. Introduction

Organoboron compounds are important synthetic intermediates for a large variety of transition metal-catalyzed C–C and C–X (X: O, N, S) bond-forming reactions [1,2]. These types of reactions generally exhibit high yields and are compatible with a large variety of functional groups, allowing the selective formation of new C–C bonds in highly functionalized substrates. More specifically, cross coupling reactions are compatible with carbonyl groups that are generally unreactive under the conditions required for the coupling of boronate moieties. One of the easiest ways to obtain organoboronates is the use of diborane as a reagent in the presence of suitable catalysts [3,4]. Depending on the reaction conditions and catalysts, diboranes can provide electrophilic and nucleophilic boron species that are able to form C–B bonds [5].

The conjugate addition of B_2_pin_2_ reagent to α,β-unsaturated carbonyl compounds is a convenient strategy to prepare functionalized organoboron compounds through the incorporation of a Bpin unit at the β-position of the carbonyl group [6]. This organic transformation was reported with a series of homogeneous catalysts consisting of transition metals such as Pt [7,8], Rh [9], Cu [10,11,12,13], Ni [14], N-heterocyclic carbenes [15,16], and Brönsted bases [17]. In this regard, it has been recently reported that 2-cyclohexenone can react with B_2_pin_2_ in the presence of metallic Cu, Cu(I) or Cu(II) salts or complexes to obtain β-ketopinacolboronates [18,19,20,21,22,23]. Although homogeneous copper salts or complexes have been reported for this reaction [23], examples on the use of heterogeneous copper-based catalysts are still limited.

**Scheme 1:**
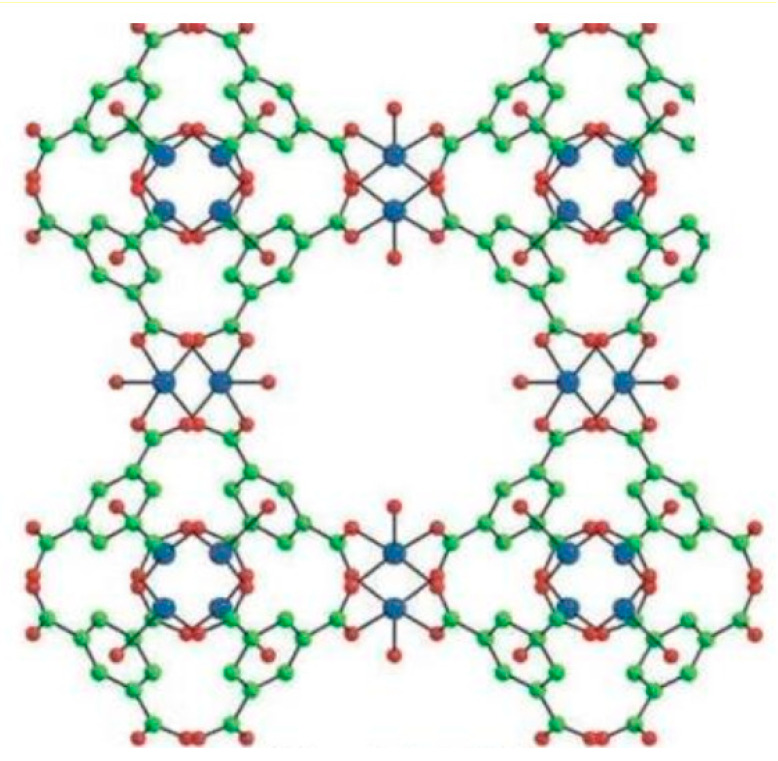
Structure of Cu_3_(BTC)_2_. Reproduced with permission from [25]. Copyright 2015 Elsevier.

Cu_3_(BTC)_2_ (BTC: 1,3,5-benzenetricarboxylate) is a type of metal organic framework (MOF) whose metal nodes are constituted by dimeric Cu^2+^-ions with octahedral coordination positions around each Cu^2+^ ion that are satisfied by four carboxylate groups of different BTC linkers, a Cu-Cu bond and a solvent molecule, typically N,N’-dimethylformamide (DMF), employed during the synthesis. This solvent molecule can be easily removed by thermal treatment under vacuum without damaging the crystal structure, generating coordinatively unsaturated positions around Cu^2+^ that are able to activate substrates and reagents [24]. The structure of Cu_3_(BTC)_2_ is shown in Scheme 1 [25]. These metal nodes and linkers define large cavities in a highly open structure with a high surface area (~1300 m^2^/g) and porosity (1.6 nm). Among the various MOFs that have been tested in heterogeneous catalysis to promote organic transformations [26,27], Cu_3_(BTC)_2_ is one of the MOFs frequently used for a broad range of reactions due to the presence of coordinatively unsaturated metal sites around Cu^2+^ ions. These Cu^2+^ ions with a coordination free position behave as Lewis acid sites in a heterogeneous fashion, thus providing opportunities to replace homogeneous Lewis acid catalysts for organic reactions. Therefore, Cu_3_(BTC)_2_ has been widely used as a catalyst to promote a variety of Lewis acid-catalyzed organic reactions including condensation [28], the ring opening of epoxide [29], and cyclizations [30,31,32], among others [33,34,35]. Furthermore, Cu^2+^ ions in Cu_3_(BTC)_2_ MOF with unsaturated positions have also been reported in many organic transformations like the oxidation of styrene [36], aerobic epoxidation of olefin [37], CO oxidation [38], oxidative synthesis of quinazolinones [39], synthesis of tetrazoles [40], arene borylation [41], Friedel–Crafts alkylation of indoles with nitroalkenes [42], dehydrogenative coupling of dimethylphenylsilane with phenol [43], acetalization of benzaldehyde [44], aldol synthesis of pyrimidine-chalcone hybrids [45], and hydrogenation of acetophenone by silanes [46].

Considering the broad scope of Cu_3_(BTC)_2_ in heterogeneous catalysis [47,48,49], one general tendency in catalysis is to develop solid and recoverable catalysts for those processes that are carried out using soluble homogeneous catalysts. Herein, it is reported that Cu_3_(BTC)_2_, a commercially available MOF, is a suitable and stable solid catalyst to promote the formation of β-keto organoboranes. We wish to make use of the active Cu^2+^ ions in Cu_3_(BTC)_2_ as catalytically active sites for this transformation. The observed results in this work are highly promising due to the fact that the catalyst is readily available as well as the fact that it can be easily reused in consecutive cycles.

## 2. Materials and Methods

### 2.1. Materials

Cu_3_(BTC)_2_ was purchased from Sigma Aldrich with the commercial trade name Basolite C300. Similarly, other related MOFs like Al(OH)(BDC) (BDC: 1,4-benzenedicarboxylate) and ZIF-8 with the trade names Basolite A100 and Basolite Z1200, respectively were also purchased from Sigma Aldrich, Barcelona, Spain. MIL-101(Cr) was synthesized by adopting earlier procedure, and its structural integrity was confirmed by powder X-ray diffraction (XRD), which was in good agreement with an earlier report [41]. Furthermore, CuCl, Cu(NO_3_)_2_.3H_2_O, Cs_2_CO_3_, K_2_CO_3_, Na_2_CO_3_, B_2_pin_2_, 2-cyclohexenone and acetonitrile were procured from Sigma Aldrich, Barcelona, Spain.

### 2.2. Experimental Procedure

In a typical catalytic reaction, a dry two-neck flask was charged with 0.5 mmol of 2-cyclohexenone and 0.5 mmol of B_2_pin_2_ followed by the addition of a base (0.12 mmol). To this mixture, 40 mg of Cu_3_(BTC)_2_ (0.0066 mol%) was added, followed by dilution with 2 mL of acetonitrile. Later, this reaction mixture was immersed in a preheated hot plate maintained at the required temperature. The progress of the reaction was monitored by sampling the aliquots at different time intervals. These samples were analyzed by gas chromatography (GC) using the internal standard method. Furthermore, the reaction mixture was also analyzed by gas chromatography coupled with mass spectrometry (GC-MS) to confirm the formation of the products. Reusability experiments were performed by recovering the solid from the reaction mixture, washed with acetonitrile and dried at 100 °C for 3 h. This catalyst was used with the fresh reactants, following an identical procedure to the one described above. On the other hand, control experiments with homogeneous soluble catalysts and radical quenchers like 2,2,6,6-tetramethyl-1-piperidinyloxyl (TEMPO), 2,6-di-*t*-butyl-4-methylphenol were also performed under identical conditions to the ones described above with appropriate loading, as mentioned in Table 1.

### 2.3. Product Analysis

The progress of the reaction was monitored by sampling aliquots (100 μL) at various reaction times. These aliquots were diluted by adding 2 mL of acetonitrile before filtering through a Nylon membrane (0.2 μm), and the resulting samples were injected in GC using a flame ionization detector with the following conditions: methyl-phenyl silicone column, TRB-5MS; 30 m 0.32 mm 0.25 μm; initial column temperature of 30 °C; initial holding time of 3 min, temperature ramp rate of 10 °C/min and final temperature of 280 °C. Quantification was done by calibration plots to obtain the relative response factors (RF) of the products as well as starting materials compared to the internal standard (nitrobenzene). The final reaction mixture was analyzed by Agilent 5973 GC-MS (Madrid, Spain) to confirm the product mass and fragmentation.

Metal leaching from Cu_3_(BTC)_2_ under the present experimental conditions was also checked by inductively coupled plasma optical emission spectrometry (ICP-OES) analysis. Briefly, the solid catalyst was removed by filtration using a 0.2 μm Nylon filter after the final reaction time. Then, this organic phase was mixed with 30 mL of 3 M aqueous nitric acid and stirred for 20 h at 80 °C. Later, the aqueous phase was separated and analyzed by ICP-OES to determine Cu in the final reaction mixture.

Furthermore, the conversion of 2-cyclohexenone was determined using the following formula.

RF was obtained with the following equation:

RF = Area of nitrobenzene moles of 2-cyclohexenone/Area of 2-cyclohexenone moles of nitrobenzene

The moles of the remaining 2-cyclohexenone and the product were calculated using the following equation:

Moles of 2-cyclohexenone = RF × moles of nitrobenzene × Area of 2-cyclohexenone/Area of nitrobenzene

Conversion (%) = moles of 2-cyclohexenone reacted/initial moles of 2-cyclohexenone

## 3. Results and Discussion

**Figure 1:**
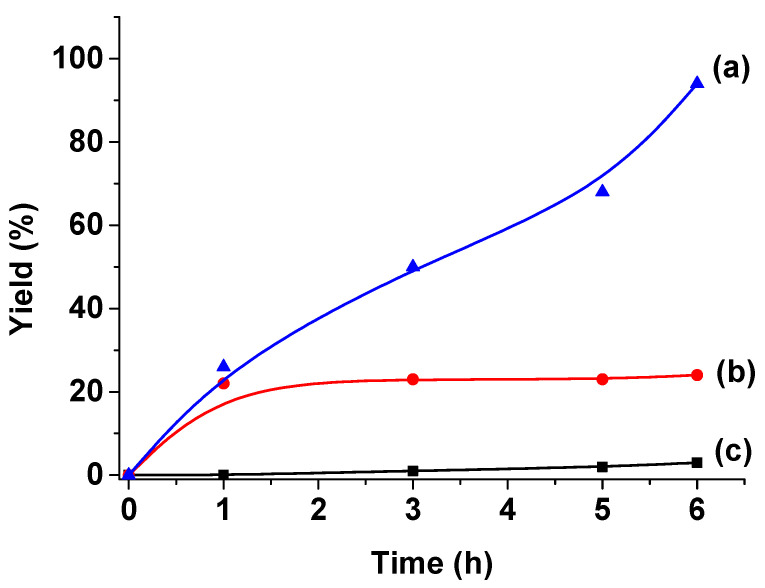
(a) Time yield plot for the formation of the desired product, (b) hot-filtration test at 1 h reaction and (c) the corresponding blank control experiment in the absence of the catalyst under identical reaction conditions.

As mentioned in the introduction, the main objectives of this work are to develop a benign and convenient heterogeneous catalyst based on cost-effective Cu metal as an active site to promote the addition of B_2_pin_2_ to enones. Hence, 2-cyclohexenone was selected as a model substrate reacting with B_2_pin_2_ as the borylating reagent to optimize the activity of Cu_3_(BTC)_2_ as a solid catalyst under different conditions. The observed results are summarized in Table 1. The β-boronation of 2-cyclohexenone did not occur either in the absence of Cu_3_(BTC)_2_ or in the absence of cesium carbonate as a base both at room temperature or at 60 °C (Entries 1–2, Table 1). In contrast, the combination of Cu_3_(BTC)_2_ and cesium carbonate afforded the desired product of 80% yield after 24 h at room temperature (Entry 3, Table 1). The reaction was faster and afforded a higher product yield at a 60 °C reaction temperature after 6 h (Entry 6, Table 1), while the relevant control experiments at 60 °C showed almost no reaction (Entries 4–5, Table 1). The product yield was almost unaffected when the reaction was carried out under inert atmosphere or using potassium carbonate as a base (Entries 7–8, Table 1). This means that ambient oxygen is not playing any role and that other carbonates can promote borylation equally. It was, however, observed that the use of sodium carbonate led to a significant decrease in the product yield (Entry 9, Table 1). A comparison of the catalytic activity under the present conditions with either Cu(I) or Cu(II) salts or with other MOFs such as MIL-101(Cr), MIL-53(Al), ZIF-8 showed either no products were formed or the yields were much lower than that achieved with Cu_3_(BTC)_2_ (Entries 10–14, Table 1). These results clearly indicate that this reaction is promoted efficiently with Cu^2+^ as active sites, while other MOFs with Cr^3+^ and Zn^2+^ are ineffective for this transformation under these reaction conditions. These results are also in agreement with a previous report where the C–H borylation reaction of arene with B_2_pin_2_ was promoted by Cu_3_(BTC)_2_ while other MOFs failed to provide the desired product [41]. Although enantioselective borylation of 2-cyclohexenone with B_2_pin_2_ was reported by CuCl using NaOt-Bu in MeOH/THF at a 92% yield with 98% ee [18], CuCl in the presence of cesium carbonate gives less than one half of the yield achieved with Cu_3_(BTC)_2_ in the present work. Deactivation seems to be the main reason for the lower yield of the homogeneous catalyst, since the reaction in the presence of the homogeneous catalyst stops after 1 h, giving a 41% yield, and after this time the reaction does not progress further for longer reaction times. In contrast, the temporal profile of product formation in the case of Cu_3_(BTC)_2_ as a catalyst shows a gradual increase in the product yield over time until a very high product yield is achieved (Figure 1).

**Table 1:** Borylation of 2-cyclohexenone using B_2_pin_2_ under various reaction conditions ^a^.

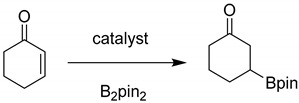					
Run	Catalyst	Base	T (°C)	Time (h)	Yield (%) ^b^
1	-	Cs_2_CO_3_	RT	24	-
2	Cu_3_(BTC)_2_	-	RT	24	-
3	Cu_3_(BTC)_2_	Cs_2_CO_3_	RT	24	80
4	-	Cs_2_CO_3_	60	6	3
5	Cu_3_(BTC)_2_	-	60	6	-
6	Cu_3_(BTC)_2_	Cs_2_CO_3_	60	6	94
7	Cu_3_(BTC)_2_	Cs_2_CO_3_	60	6	92 ^c^
8	Cu_3_(BTC)_2_	K_2_CO_3_	60	6	92
9	Cu_3_(BTC)_2_	Na_2_CO_3_	60	6	55
10	CuCl	Cs_2_CO_3_	60	1, 6	41, 41 ^d^
11	Cu(NO_3_)_2_·3H_2_O	Cs_2_CO_3_	60	6	- ^e^
12	MIL-101(Cr)	Cs_2_CO_3_	60	6	6
13	Al(OH)(BDC)	Cs_2_CO_3_	60	6	-
14	ZIF-8	Cs_2_CO_3_	60	6	5
15	Cu_3_(BTC)_2_	Cs_2_CO_3_	60	6	82 ^f^
16	Cu_3_(BTC)_2_	Cs_2_CO_3_	60	6	88 ^g^

^a^ Reaction conditions: 2-cyclohexenone (0.5 mmol), B_2_pin_2_ (0.5 mmol), base (0.12 mmol), catalyst (40 mg), CH_3_CN (2 mL); ^b^ Determined by GC; ^c^ Reaction performed under inert atmosphere; ^d^ 20 mg of CuCl; ^e^ 48 mg of Cu(NO_3_)_2_·3H_2_O; ^f^ 50 mg of 2,6-di-*t*-butyl-4-methylphenol; ^g^ 50 mg of TEMPO.

One of the essential experiments to be performed in heterogeneous catalysis is the analysis of the metal leached from the solid to the liquid phase (leaching analysis) or a hot-filtration test to prove the stability of a heterogeneous catalyst under the optimized reaction conditions. Regarding this aspect, a hot-filtration test was conducted under optimal reaction conditions, as shown in Table 1. The solid catalyst was removed after a 1-h reaction time while the mixture was hot when the product yield was about 25%. The kinetic profile of this test indicates that the reaction completely stops upon removal of the solid catalyst from the reaction mixture, thus providing sound evidence in support of the operation of heterogeneous catalysis (Figure 1). On the other hand, the possibility of metal leaching to the reaction mixture is also ruled out since the ICP-OES analysis indicated <1 ppm of Cu in the final reaction mixture, thus supporting the operation of heterogeneous catalysis.

**Figure 2:**
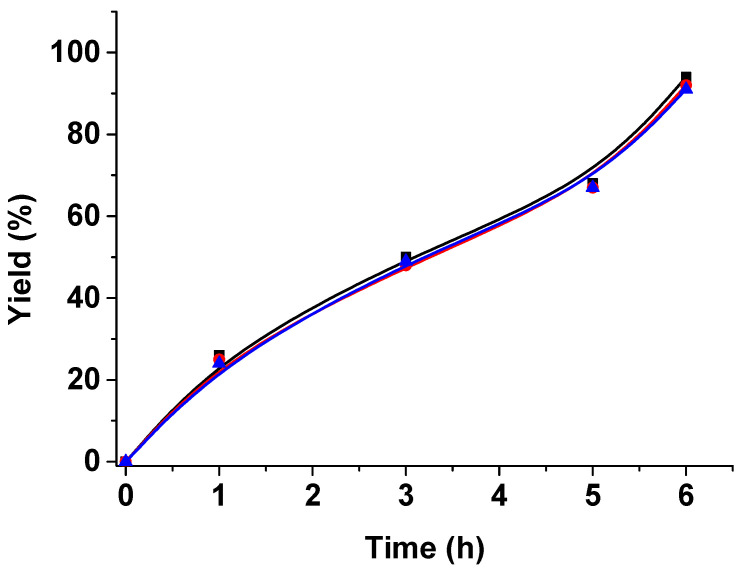
Temporal profile for the fresh (■), 1st reuse (●) and 2nd reuse (▲) of Cu_3_(BTC)_2_ during the reusability test.

**Figure 3:**
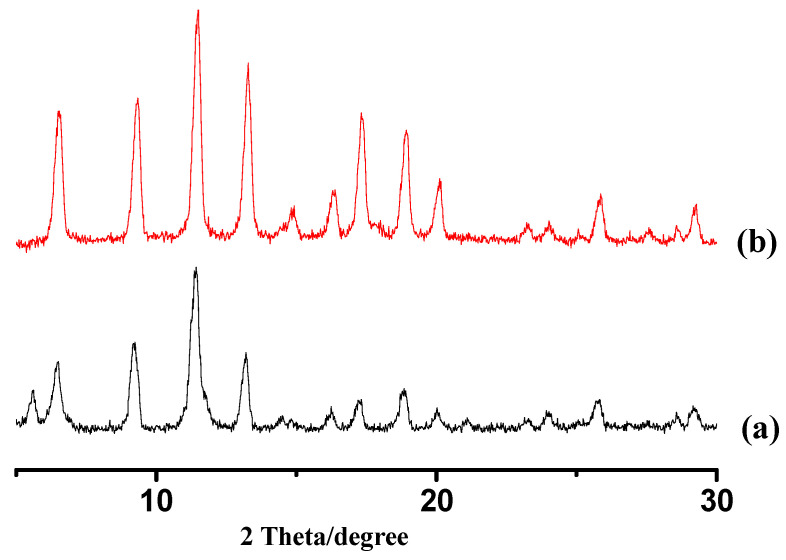
Powder XRD patterns of (a) fresh and (b) twice reused Cu_3_(BTC)_2_ solids.

Another established procedure to ascertain catalyst stability is to monitor the activity of the recovered solid in consecutive cycles, often referred to as a reusability test. Hence, Cu_3_(BTC)_2_ was recovered at the end of the reaction, washed and dried at 100 °C to perform a consecutive borylation reaction. The experimental results showed that the temporal profiles (Figure 2) as well as the final yields were not altered up to two recycles. The product yields for the fresh, first and second reuses were 94, 92 and 91% under the conditions shown in Table 1 (entry 6). The powder XRD of the twice reused solid showed a decreased peak intensity around 21° with the appearance of a new peak at 15° compared to the XRD patterns for the fresh solid (Figure 3). These changes in the peak intensity are possibly due to the adsorption of organic products; however, the crystallinity of the reused solid is retained. On the other hand, the Cu content of the fresh and twice reused Cu_3_(BTC)_2_ was 28.27 and 28.25%, respectively, suggesting an almost similar Cu content in the reused material. Furthermore, FT-IR spectroscopy was used to characterize Cu_3_(BTC)_2_ before and after catalysis. FT-IR spectra of the fresh Cu_3_(BTC)_2_ showed a characteristic asymmetric stretching vibration at around 1680 cm^−1^ due to the carboxylate group in the BTC linker. Additionally, symmetric stretching vibrations were observed at around 1424 and 1360 cm^−1^. These characteristic spectral features were also observed in the twice reused Cu_3_(BTC)_2_ without observing any additional bands. These results clearly indicate the retainment of structural integrity and that Cu_3_(BTC)_2_ is highly stable under the present experimental conditions.

In an earlier precedent, Kobayashi and co-workers proposed a reaction mechanism involving the cleavage of the B-B bond by the base, forming pinacolyl borate and a nucleophilic boronate species that becomes coordinated to the Cu site [19]. Subsequently, the coordination of α,β-enone to the Cu atom and transfer of the Bpin moiety from the Cu^2+^ ion to the β-carbon would render the final product and would restore the catalytically active Cu species. It is likely that a similar reaction mechanism could be operating in the present case. In support of this proposal, FT-IR spectroscopy of Cu_3_(BTC)_2_ after the incorporation of B_2_pin_2_ shows spectroscopic changes compatible with the formation of Cu-Bpin and the weakening of the Cu-Cu bond [41]. These spectroscopic signatures are reversed upon the desorption of Bpin by thermal evacuation under vacuum [41]. Additionally, the fact that the product yield is not affected by the presence of 2,6-di-*t*-butyl-4-methylphenol (Entry 15, Table 1) and TEMPO (Entry 16, Table 1) that are typical C-centred radical quenchers, thus ruling out the reaction mechanism involving carbon radicals.

**Scheme 2:**
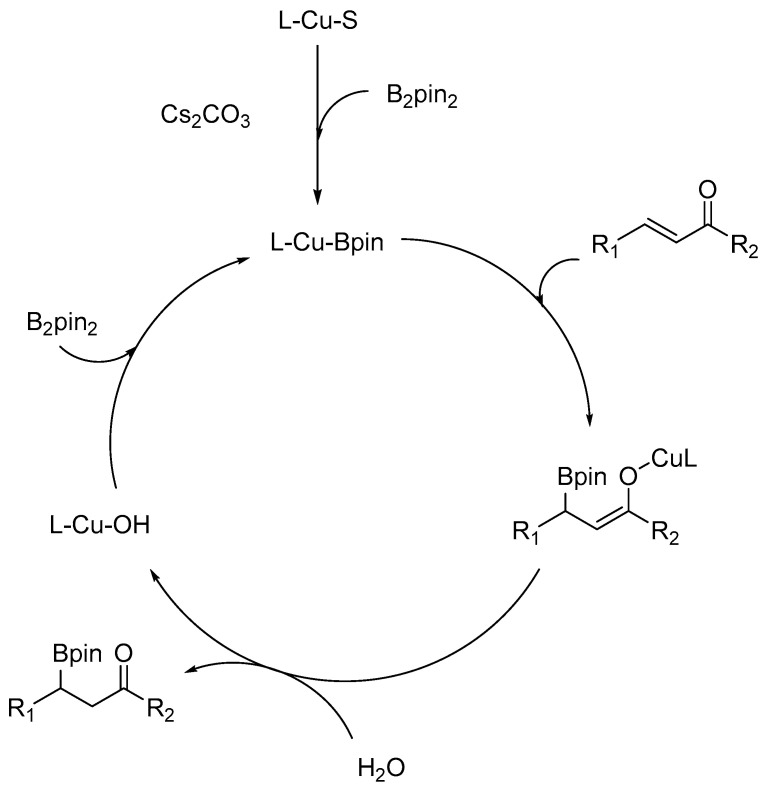
Proposed mechanism for the conjugate addition of B_2_pin_2_ to α,β-unsaturated carbonyl compound in the presence of Cu_3_(BTC)_2_. L represents BTC linkers; S indicates solvent or water.

Considering the available catalytic data and the results obtained by quenching experiments, the following mechanism is proposed for this transformation. The crystal structure of Cu_3_(BTC)_2_ contains water molecules that are loosely bound to the coordinatively unsaturated sites around copper atoms [50]. Initially, these coordinatively unsaturated metal sites in Cu_3_(BTC)_2_ react with B_2_pin_2_ in the presence of a base to afford reactive copper-boryl intermediates, which behave as the catalytically active species. Later, these copper-boryl complexes promote the conjugate addition to the α,β-unsaturated carbonyl compound to provide an organocopper intermediate, which upon hydrolysis with water affords the desired product by generating a copper hydroxide. This reacts further with B_2_pin_2_ to regenerate the active copper-boryl complex species. This mechanistic proposal is depicted in Scheme 2.

As mentioned earlier in the introduction, wide ranges of catalytic systems have been developed for the borylation of organic compounds using mostly homogeneous and heterogeneous catalysts. Specifically, copper-based catalysts have been employed for borylation reactions with a broad spectrum of substrates. For instance, enantioselective boron conjugate addition to dienone was reported in water with Cu(OH)_2_ in a heterogeneous fashion or with Cu(OAc)_2_ as a homogeneous catalyst [51]. In another precedent, the dehydrogenative borylation of styrene with B_2_pin_2_ was reported by copper hydroxide supported over CeO_2_ or Al_2_O_3_. The product selectivity was determined by the appropriate choice of a suitable ketone and support [52]. Recently, the borylation of alkyl bromides and chlorides using B_2_pin_2_ was reported with Cu/Pd alloy nanoparticles supported over graphene under photocatalytic conditions [53]. On the other hand, a microporous MOF was synthesized with an imidazolium-containing ligand that can generate in situ NHC-CuCl units (NHC: N-heterocyclic carbenes), which can efficiently promote the addition of B_2_pin_2_ to 2-cyclohexenone [54]. A quantitative yield of the desired product was achieved at 25 °C for 24 h in the presence of MeOH and Cs_2_CO_3_ in THF as solvent. This solid retained its activity for five cycles with no decay in the yield. In contrast, the present work operates under different experimental conditions in terms of solvent, reaction temperature, substrate/catalyst loading, no use of methanol and Cu oxidation state. Hence, the present results complement the existing reactions and represent a more amenable catalytic system based on a commercial catalyst. Interestingly, these results clearly indicate that this organic transformation can be effectively promoted by Cu(I)- or Cu(II)-based MOFs instead of Pt-, Pd- and Ir-based catalysts.

**Figure 4:**
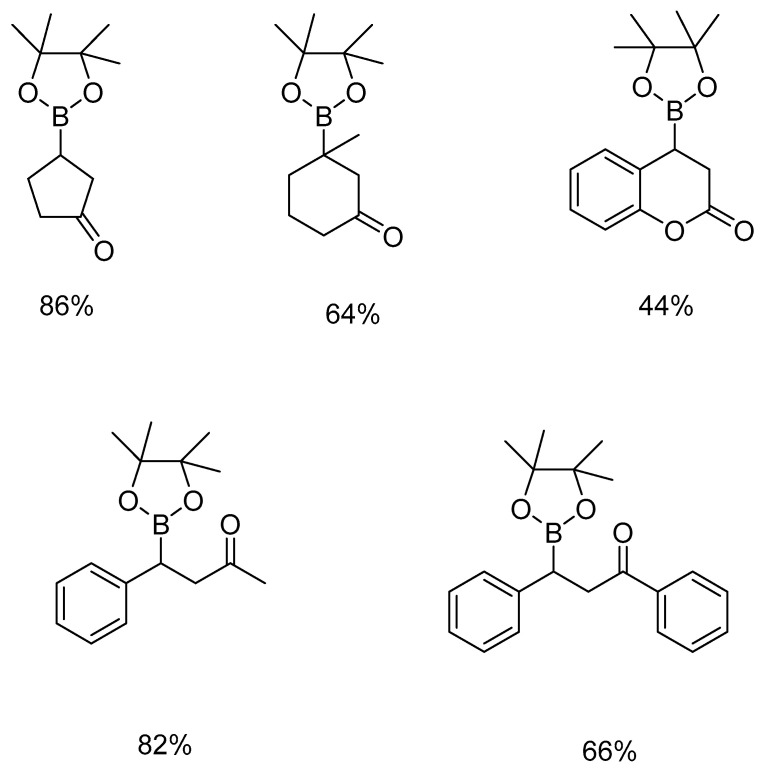
Scope of Cu_3_(BTC)_2_ for the β-boronation of various α,β-unsaturated substrates. The percentage yields of these products correspond to the GC yield. Reaction Conditions: enone (0.5 mmol), B_2_pin_2_ (0.5 mmol), Cs_2_CO_3_ (0.12 mmol), Cu_3_(BTC)_2_ (40 mg), CH_3_CN (2 mL), 60 °C, 6 h.

The scope of the reaction was screened for different substrates. The results are presented in Figure 4 where the yields of the products have also been indicated. Thus, the formation of the boronates in high yields was observed for 2-cyclopentenone (86%) and benzylideneacetone (82%). On the other hand, 3-methyl-2-cyclohexenone and chalcone also gave the expected C-B coupling products in moderate yields of 64% and 66%, respectively. Lower yields for the C-B coupling product were obtained in the case of coumarin (44%) as a substrate due to the lower reactivity of this aromatic lactone. These products were confirmed by GC-MS.

## 4. Conclusions

The present manuscript has shown that Cu_3_(BTC)_2_ in the presence of cesium carbonate as a base is a suitable solid catalyst for promoting the formation of β-keto organoboranes in high to moderate yields. The solid acts as a heterogeneous catalyst and can be reused in consecutive runs without decrease in activity. Considering the importance of functionalized organoboranes, this work represents some significant merits by employing readily available Cu_3_(BTC)_2_ without containing non-noble metal as a convenient catalyst for the synthesis of β-keto organoboranes under mild reaction conditions. Further work is required to understand the mechanistic aspects of this transformation.
